# Historical Developments and Paradigm Shifts in Public Health Nutrition Science, Guidance and Policy Actions: A Narrative Review

**DOI:** 10.3390/nu11030531

**Published:** 2019-02-28

**Authors:** Ella Ridgway, Phillip Baker, Julie Woods, Mark Lawrence

**Affiliations:** 1 School of Exercise and Nutrition Sciences, Deakin University, Geelong 3220, Australia; emrid@deakin.edu.au; 2 Institute for Physical Activity and Nutrition (IPAN) School of Exercise and Nutrition Sciences, Deakin University, Geelong 3220, Australia; j.woods@deakin.edu.au (J.W.); lawrence@deakin.edu.au (M.L.)

**Keywords:** public health nutrition, nutrition science, dietary guidelines, environmental sustainability, paradigm shifts

## Abstract

Public health nutrition (PHN) seeks to protect and promote the nutrition-related health and wellbeing of populations. PHN science is dynamic and has evolved over time, helping to inform our understanding of the changing nature, scope, causes and solutions to PHN problems. This scientific basis has informed nutrition guidance and policy. Using a narrative synthesis method and guided by Kuhn’s theory on the structure of scientific revolutions, this paper reviews the historical development of PHN, aiming to understand the emergence of major scientific paradigms, paradigm shifts and evidence-informed guidance and policy. We propose that the development of PHN is characterized by the successive layering of paradigms resulting from interactions between science, social change and policy-making. Four eras of PHN are evident: the foundation, nutrient deficiency, dietary excess and imbalances, and environmental sustainability (ES). Dominant paradigms have been communicated through nutrient reference standards, dietary goals and dietary guidelines. Transitions from one era to the next indicated new ways of thinking about PHN, amounting to a paradigm shift. The bidirectional relationship between nutrition and ES is the latest challenge confronting PHN. Investigating PHN paradigm transitions reveals how we have arrived at current guidance and policies, and how PHN might progress into the future.

## 1. Introduction

Public health nutrition (PHN) seeks to protect and promote the nutrition-related health and wellbeing of populations through the organised efforts and informed choices of society [1]. As a field it is grounded in health promotion, primary prevention and public health, as distinct from basic, biomedical or metabolic nutrition [2]. Contemporary approaches to PHN integrate a food and nutrition systems focus with wider social, cultural, economic and environmental co-determinants and solutions, and human rights principles [3]. The generation, synthesis and translation of scientific evidence obtained from studies investigating the relationships between nutrients, foods, dietary patterns and health outcomes into nutrition guidance is a core function of PHN [4]. In this study, PHN guidance refers predominantly to nutrient reference values (NRVs), dietary goals and dietary guidelines designed to inform policy and regulatory actions. Such guidance is typically produced by food and public health authorities operating at international and national levels in the form of policy documents, discussion papers, technical reports and dietary guidelines.

Rapid and profound social, cultural, economic and technological changes have contributed to the emergence of new and unprecedented challenges for PHN [5]. These include the global spread of obesity and non-communicable diseases alongside persistent undernutrition and food insecurity, with many individuals, households and populations now experiencing a ‘double burden’ of malnutrition [6,7]. Increasingly environmental sustainability (ES), as mediated by food system activities [8,9], has become one of the most urgent challenges of the 21st century [3,10,11]. Of particular concern are the effects of climate change, land degradation, ecosystem and biodiversity loss, the overreliance of current food production practices on fossil fuels and finite natural resources [12,13]. Many countries are also undergoing a nutrition transition—a shift from traditional to western dietary patterns higher in animal-sourced foods, cheap vegetable oils and highly-processed energy-dense foods. Such diets are associated with higher environmental impacts compared with plant-based and whole food alternatives [14,15].

It is against this backdrop that the revision and development of PHN guidance must now be revisited. The purpose of this review is to describe the historical development of PHN and the scientific basis of current PHN guidelines, thereby tracing the emergence of and shifts in PHN paradigms. A paradigm is a concept that refers to a distinct set of beliefs and values used to order and explain the complexity of a given phenomenon, including a set of ontological, epistemological and theoretical assumptions that determine problem definitions and solutions, acceptable methodologies, methods and tools that generate evidence, establish authoritative sources of information, and legitimise actors and institutions [16,17,18,19]. Paradigms hence function as shared intellectual and normative frameworks that orientate groups and individuals working within a particular discipline or policy system. Therefore, a paradigm shift occurs ‘when the usual way of thinking about or doing something is replaced by a new and different way’ as a result of fundamental changes in approach or to the underlying assumptions described above [16]. Nutrition science paradigms inform our understanding of the nature, extent, causes and possible solutions of PHN problems.

Previous studies have undertaken an historical analysis to examine the development of PHN [20], nutrition science [21,22,23,24,25,26], and nutrition guidance [27,28,29,30,31]. Santich also considers paradigm shifts in dietary advice [32], drawing on Kuhn’s ‘structure of scientific revolutions’ to explain the evolution of dietary guidance in Australia [16]. This review focuses on the contextual and ideational aspects of paradigms and paradigm shifts, including the underlying ontology and epistemology of the nutrition science discipline that underpins PHN policy and practice by drawing on the theories of Kuhn. It is the first to consider the effect of the ES challenge on nutrition guidance development. 

This work is timely, as the debate around the development of dietary guidelines has become highly contested with respect to the inclusion of ES considerations. There is a growing evidence base to suggest that dietary patterns with low environmental impacts are consistent with good health [8,14,33,34,35]. However, only five countries (Brazil, Canada, Sweden, Qatar and Germany) have successfully integrated ES considerations into their dietary guidelines. Two others (Australia and the United States) have made attempts but failed to achieve government endorsement [35]. In investigating the PHN science paradigm transitions of the past, we aim to learn how we have arrived at current dietary guidance recommendations and understand how they might progress in the future in the context of ES considerations. 

## 2. Methods

### 2.1. Study Design

This paper adopts a theoretically-guided narrative literature review and synthesis method [36,37]. This method was selected because it allows for the integration and interpretation of qualitative findings from a diversity of studies on a broad and multi-faceted research topic. The theoretical component is important, because it enables a guided understanding of the history and development of the phenomenon under study [38]. This involved three steps: first, the explication of theory to guide the search and analysis; second, a search for relevant literature; and third, analysis of the final documentary sources and thematic synthesis of the results.

### 2.2. Theory

The development of PHN guidance occurs at the intersection of PHN science and policy-making. We draw upon Kuhn’s *Structure of Scientific Revolutions* [16], a landmark theory of paradigm shifts widely regarded as an influential contribution to the philosophy of scientific knowledge. According to Kuhn, periods of “normal science” are interrupted by periods of “revolutionary science” leading to a paradigm change. Science progresses ‘accumulatively’ through long periods of conceptual continuity as scientific methods and measures, which are “selected…only because they promise opportunity for the fruitful elaboration of an accepted paradigm” ([16], p. 126). Anomalies and new scientific discoveries lead to scientific revolutions when the existing theory can no longer explain observations or research findings. A new paradigm that more adequately explains the growing set of anomalies and sets out a new model for problems and solutions within the discipline is proposed. In other words, “when communities of experts fail to provide further scientific advance, they are overtaken and replaced by other communities with different ideas and ways of thinking…caused by a crisis prompted by new information” ([39], p. 90). Kuhn explains that there is initial denial or resistance to the new paradigm but as experiments, instruments and literature increases, more scientists adopt the new paradigm and a period of normal science returns under its dominance. As such, Kuhn’s notion of paradigm is inherently political, making it appropriate for examining conceptual models of political phenomena [40]. Kuhn proposes five phases of a paradigm shift, as outlined in Table 1. 

### 2.3. Search Strategy 

*Google Scholar* was selected as a broad, comprehensive database for the literature search and was supplemented with *PubMed* because of its widespread use and relevance to the PHN discipline. In June 2018 both databases were searched with no date limits applied. Search terms included (nutrient reference standard* OR nutrient reference value* OR recommended nutrient intake*; dietary guideline* OR food-based dietary guideline*) and (history OR development OR evolution) with * indicating variants of the term. We applied a citation snowballing technique to the *Scopus* records of key authors and reference lists of sampled articles. Titles and abstracts of all sources were screened. Full-texts were retrieved and reviewed on occasions where the title and abstract lacked sufficient detail. We then applied a quasi-historical approach to trace major PHN meetings and reports, as well as grey literature pertaining to nutrition science knowledge, political, social and economic events. Google Advanced search was used to systematically search for grey literature from the Food and Agricultural Organization (FAO) and World Health Organization (WHO) using the ‘site or domain’ function to narrow the results. Case studies outlining the process of developing a particular iteration of national-level nutrition guidance were excluded from this review.

### 2.4. Analysis/Synthesis

This review considers the historical development of and paradigm shifts in PHN and nutrition guidance in a broadly chronological fashion. From here, data was synthesised into a series of phases associated with an approximate period of time and in reference to the dominant PHN challenge of the day. Our analysis pays particular attention to synthesising the transitions and relationships between each period as a result of emerging ideas about nutrition and health, therefore resulting in the corresponding evolution of nutrition guidance from one period to the next. 

## 3. Results

Public health nutrition science and guidance have progressed through a series of four main eras, as a result of the evolving body of evidence for nutrition problems, in response to rises and falls in the prevalence of nutrition-related diseases and problems, and by major shifts in thinking about nutrition and health. We refer to these as the foundation, nutrient deficiency, dietary excess and imbalances, and food system sustainability eras. These labels indicate the main PHN challenges characterizing each era. We propose that each of these eras broadly relates to the phases outlined by Kuhn and that, as a discipline, PHN is currently in the process of a scientific revolution. A summary of each era is provided in Table 2. 

The dominant paradigms of each era were communicated through the development of three systems of nutrition guidance: NRVs, dietary goals and dietary guidelines. The nature, scope and purpose of each is outlined in Table 3. Although they have emerged sequentially, the transition from one prominent PHN challenge, and system of nutrition guidance, to the next has enabled each of the eras to overlap. In this sense, the end of one era does not signal the immediate commencement of another. While they can be minimized, the threats to the nutrition-related health and wellbeing of populations can never be completely eradicated. Therefore, all four eras coexist as ‘layers’ to varying degrees. For example, dietary guidelines traditionally mix food and diet epidemiology, whilst still incorporating nutritional adequacy objectives. Gradually the scope of an increasing number of national dietary guidelines is extending to include ES considerations.

### 3.1. Foundation Era

According to Kuhn, a “pre-paradigm” phase occurs only once in the beginning of a scientific discipline and is characterised by incomplete or incompatible theories in the absence of a common body of knowledge to draw on. Religious and philosophical writings have included early forms of nutrition guidance for more than 6000 years [26,32]. However, the foundations of modern nutrition science did not emerge until the 18th century. The first PHN intervention was recorded in 1753, when Lind recommended the inclusion of citrus fruits in the rations of British navy sailors to prevent scurvy on long sea voyages [45]. This work provides an early example of the way that governments can intervene on the nutritional health of populations for national benefit—in this instance, to increase the exploration and fighting capacities of naval forces [42]. However, an understanding of the biochemical nature of food and nutrition was not possible until the Chemistry Revolution in France during the late 1700s. In keeping with the dominant explanations for disease causation (miasma theory, implicating ‘poisonous vapours’), it was believed that citrus fruits worked by countering the ‘negative effects of sea air’ [21].

Advances in the methodology of chemistry provided early explanations of the respiratory process, metabolism, energy production and calorimetry [26,46,47]. Refining substances, including food, into smaller and smaller constituents was central to the nature of chemistry, and eventually for nutrition science. Nitrogen was a key focus for many scientists and their experiments—it was identified as a primary chemical component of meat and was considered essential for health [48]. Working from Giessen in Germany, von Liebig (an early founder of biochemistry) recognised protein and its important physiological role in the growth of animals and humans. Subsequently, protein was heralded as the ‘true’ or ‘master’ nutrient [21,26]. With the arrival of the Industrial Revolution, von Liebig and his colleagues realised that the increasingly mechanised food system could be engineered towards increased production and consumption of protein, transforming the meat and dairy industries. This emphasis on protein and animal products has remained an enduring ideology of food systems [26]. 

The Industrial Revolution also encouraged widespread migration towards cities, resulting in overcrowding, hunger and poor sanitation. Outbreaks of communicable diseases spread rapidly under these conditions, particularly cholera. This became a major impetus for governments to develop legislation on sanitation and to improve living conditions in support of public health [49]. Miasmic theory was ultimately invalidated by the germ theory of disease proposed by Pasteur, Koch and others by the end of the 1880s [50]. Here, disease is caused by microorganisms and other contaminants in food and water [51]. Hence, food safety became a significant public health priority as a result of sanitation efforts to combat pathogenic disease. Nutrition science extended to the development of food processing techniques and methods like pasteurisation to reduce the risk of food-borne illness [27]. Early food policies sought to protect public health by addressing food safety and adulteration [52]. In addition, education programs and early institutional frameworks for PHN emerged during this time, namely the founding of ministerial departments of health [20,53]. The incidence of food-borne illnesses declined dramatically as a result of these measures, alongside increasing recognition of the wider benefits of public–private collaboration and the responsibilities of governments and their agencies to improve food safety [27,49]. These early developments allowed PHN to transition to a new era and the emergence of official nutrition guidance. 

### 3.2. Nutrient Deficiency Era

According to Kuhn, in the transition from Phase 1 to 2, a dominant paradigm develops as actors begin to form a consensus around conceptual frameworks, terminologies, methodologies and modes of inquiry. “Normal science” begins under the dominant paradigm and will continue as long as there is consensus within the discipline. Anomalies that are difficult to explain under the current paradigm may occur but are usually resolved.

The global malnutrition burden in the first half of the 20th century was characterised by hunger and micronutrient deficiencies (particularly beriberi, scurvy and pellagra), thought to be infectious or pathogenic in nature. In 1912, Funk isolated a substance (vitamin B_3_ or niacin) previously shown to be essential in the diet for the prevention of beriberi. The compound contained an amine group and he coined the term “vital amine” or “vitamine”. As the structure and function of micronutrients were revealed and found not to include amines, spelling was later amended to “vitamins”. Funk therefore proposed that such diseases were caused by the deficiency of these essential substances in food [54]. Vitamin theory provided a scientific basis for the success of Lind’s scurvy intervention more than 150 years prior. This represented a significant and challenging shift in thinking for scientists and set forth ‘the newer knowledge of nutrition’ consistent with a new nutrition paradigm [55]. Vitamins became the primary focus of nutrition research over the next 30 years [23]. By the mid-20th century, all major vitamins (A, B complex, C, D, E, and K) had been isolated and synthesised [25].

In order to meaningfully influence population health outcomes, vitamin theory needed to be endorsed by governments and industry [26]. The achievements of food and nutrition scientists during the 1930s and 1940s shifted the focus from whole foods to nutrients. Micronutrient deficiencies gained particular prominence after the declaration of World War I, as many young men were rejected from service for health reasons [20]. Promoting the consumption of vitamin- and mineral-rich “protective foods” (especially milk, meat and eggs) and “balanced diets” became key messages for PHN campaigns through the 1920s and 1930s [56]. World War II posed a significant threat to food security as a result of actual and potential food shortages. Governments began to harness nutrition knowledge for utilitarian purposes [26]. Increasingly, food policies engineered the food system towards mass production methods, while food policies granted greater access to international and domestic markets, and the ‘integration of agrochemical and food manufacturing interests’ [52]. This approach has been described as the ‘productionist’ model [57]. Nutrition policies emphasised the importance of adequate population nutrition by focusing on food rations and fortification [20].

In parallel to the productionist model, and in many ways reinforcing it, a ‘reductionist’ approach to nutrition science emerged as the traditional epistemological model and has been dominant ever since [58]. As Zeiesel et al. have stated, the primary objective of reductionism in nutrition science is to explain the diet–health relationship as the sum of its parts by identifying “the molecules involved in biological events, examining them in purified forms or in simple systems” ([59], p. 1). From the reductionist point of view, diet is reduced to food groups, food items, and food constituents and health is perceived as purely physical in nature, reduced to multiple systems, their components, and biological markers [58]. As our understanding of food and health intersected with public policy agendas, the first system of official nutrition guidance, NRVs, were developed as quantitative estimates of human requirements for essential nutrients, expressed in weight/day. Box 1 defines NRVs, their purpose and scope.

The League of Nations established the first set of NRVs in 1937, followed by the United States (US) and Canada [29,60,61]. Early versions of the NRVs included just nine nutrients—protein, calcium, iron, thiamine, riboflavin, niacin, ascorbic acid, and vitamins A and D. As the scientific evidence base to support the quantification of individual nutrient requirements expanded, the number of recommended nutrients in NRVs increased [62]. Current editions of the NRVs include recommendations for over 30 macronutrients, vitamins, minerals and trace elements. During the 1990s and 2000s, the expert committees for NRVs in the United Kingdom (UK), Europe, Australia, Canada and the US recognised that chronic diseases and the effects of overconsumption also needed to be considered in NRVs. As a result, Upper Levels of Intake (ULs) were established. ULs set out the highest amount of daily nutrient intake that is unlikely to cause adverse health effects among healthy populations [63].

The promotion of dietary adequacy through the development of NRVs continues today. Nutrient reference values under development by the FAO and in the US continue to extend the traditional emphasis on dietary adequacy towards the prevention of non-communicable diseases (NCDs). These ‘recommended nutrient intakes with chronic disease endpoints’, or NRV-NCDs, rely on the evaluation of causal relationships between chronic diseases and associated nutrient intake responses [64,65]. Nutrient reference values reflect the best available evidence at a given point in time, but in recognition of the fact that there is “no assurance of complete coverage of all required nutrients”, their recommendations should be implemented through a balanced diet and wide variety of foods ([62], p. 3). This nutrient-centric focus forms the basis of nutrient-profiling methods for systems of food classification and of commercial strategies used by food companies to market ‘better for you’ products including the reformulation, fortification and functionalization of processed foods [66]. 

### 3.3. Dietary Excess and Imbalance Era

Kuhn postulates that in the shift from Phase 2 to 3, normal science becomes difficult when anomalies continue to accumulate over time, revealing weaknesses in the paradigm. If the paradigm cannot continue to explain these anomalies, a crisis period occurs.

The application of NRVs to PHN policy and practice led to a reduction in the prevalence of micronutrient deficiencies. However, they were predicated on the idea that excess was preferable to deficiency [67]. Obesity had by that time been recognized as a diet-related condition but was not considered as serious as malnutrition resulting from dietary inadequacies [56]. After World War II, the mechanisation of farming and improved technological capabilities of the food processing industry increased the availability and variety of foods significantly [56]. In 1951, a report from the second session of the Joint FAO/WHO Expert Committee on Nutrition declared that over-consumption of food could produce serious forms of malnutrition and was a significant problem for high-income countries with abundant food supplies [68]. The focus of nutrition science and PHN had begun to shift towards an era of dietary excess and imbalances, an anomaly inconsistent with the dominant nutrient deficiency paradigm [27,69].

Nutrition scientists began to investigate the links between the overconsumption of various nutrients and chronic diseases from the late 1940s and early 1950s, establishing a number of ambitious cohort studies that followed thousands of individuals over time. The Framingham Study identified multiple risk factors for cardiovascular disease (CVD), including elevated blood cholesterol and obesity [70]. Findings from the Seven Countries Study allowed Keys to establish the hypothesis that CVD risk was influenced by the composition of total fat, saturated fat and cholesterol in the diet [71]. This established one of the most dominant and lasting assumptions in nutrition guidance—that dietary fat was a likely risk factor for CVD and that replacing saturated fats from animal sources with vegetable oils could lower blood cholesterol levels and potentially prevent heart disease [72]. Yudkin challenged the dietary fats hypothesis by suggesting that sugar consumption could be the primary agent in CVD risk [73], but was largely ignored by the nutrition science community. Throughout the 1960s, a number of non-government health organisations published dietary recommendations to prevent CVD. While all groups agreed that the consumption of total fats should be reduced, there was limited consensus in recommendations on how best to achieve this goal [29]. The WHO concluded that more evidence was needed before PHN authorities could recommend any dietary changes [74]. The FAO and WHO subsequently returned their focus to hunger and micronutrient deficiencies [75]. Recommendations for other chronic diseases were developed in response to increasing evidence from nutrition science research. Some disease-specific dietary recommendations conflicted with those for other conditions [27,76].

Seeking to balance these competing risk factors, governments began developing dietary goals throughout the 1970s, representing quantified targets for selected macro- and micronutrients to support optimal nutritional health and prevent diet-related chronic diseases, expressed as average national intakes [27]. These are further defined in Box 2. Dietary goals extend the quantification of individual nutrients and food components in NRVs but are a precursor to dietary guidelines, which can then be provided as a way to shift population diets towards achieving the dietary goals [42]. The Dietary Goals released by the US in 1977 recommended the reduction of dietary fat consumption by 40 percent to 30 percent of total energy intake and of refined sugar from 45 percent to 10 percent of total energy intake [77]. However, the release of the goals generated widespread controversy among health professionals and the food industry due to the lack of consensus around the impact of certain food components on chronic disease risk, particularly among stakeholders with vested economic interests in food supply and production [56,75].

A broad international agenda to support the development of healthy public policy was developed during the 1970s and 1980s led by the WHO [78,79]. Dietary guidelines also appeared at this time. Norway published the first set of dietary guidelines in 1975, followed by the US in 1980 [44]. While early dietary guidelines were considered more cautious than the Dietary Goals in the US, the government was now on record as recommending that the population should eat less of the substances that had traditionally been vital for the food industry’s success [56]. Dietary profiles outlining the foods and nutrients associated with wellbeing and protection against disease were quantified and presented by UN bodies, governments, their agencies and authoritative organisations as dietary guidelines for populations. Dietary guidelines continued to focus on individual nutrients and food components, particularly the overconsumption of calories, total and saturated fat, sodium and sugar, and the underconsumption of dietary fibre [80].

By the 1990s, nutrition scientists and policymakers began to acknowledge that people eat foods and not nutrients [81]. Here, diet and health represent complex systems involving non-linear interactions of multiple foods, in turn comprised of multiple nutrients at any one time [58]. The mechanistic nature of reductionism failed to explain diverse metabolic effects on the entire organism, but “holism” offered an alternative epistemological approach for understanding the complexity of diet and health. Holism considers “the dynamic interaction of the parts’ in a system and implies that ‘a system as a whole has features not found in any one of the parts” ([58], p. 515). Subsequently, revisions of the first generation of dietary guidelines became more food-based. To improve nutrition-related health and food consumption, The Plan of Action adopted at the International Conference on Nutrition in 1992 called for the dissemination of nutrition information through food-based dietary guidelines (FBDGs) relevant and appropriate for the country’s population, including targeted guidelines for specific age groups and lifestyles. Box 3 provides a further definition [82]. Breastfeeding and ‘other sustainable’ food-based approaches were prioritised and dietary diversification through production and consumption of nutrient-rich foods, including appropriate traditional foods, was encouraged [35]. This indicated a significant departure from policies shaped solely by nutrient requirements, towards an agenda set by the public health concerns of the day [43]. In 1995, the FAO and WHO convened a joint consultation to establish the scientific basis for developing and using FBDGs [41]. The process resulting from this work begins with an analysis of the most critical public health issues that are related to diet within a given context, and outlines strategies to identify food-based approaches to address these issues [28]. These efforts were further strengthened by the Second International Conference on Nutrition, held in November 2014 [83].

Since the 1995 consultation, some low- and middle-income countries have also commenced development of FBDGs. Many of these countries face large differences in diet- and nutrition-related health outcomes between their wealthy minorities and poor majorities. Throughout this period, hunger and malnutrition began to coexist with obesity and diet-related chronic diseases in many countries and regions. This dynamic is described as the “nutrition transition” [84]. Nutrition transitions are associated with social changes and their influence on dietary patterns, including urbanisation, workforce changes and the globalisation of food systems [56,85,86]. Collectively, the second generation of dietary guidelines were more varied than the first due to the significant differences in individual country contexts (e.g., differences in nutrition status, food availability, culinary cultures, eating habits between low-, middle- and high-income countries and regions).

### 3.4. Food System Sustainability Era

In a shift from Phase 3 to 4, Kuhn posits that crises can often be resolved by normal science but that there are other times when the efforts of normal science within a paradigm will fail. Subsequently, the discipline enters a new phase where underlying assumptions of a discipline are re-examined, and a new paradigm is established.

Despite the ongoing development and review of dietary guidelines, only modest and uneven progress in reducing malnutrition has been achieved in recent decades [83]. In the 21st century, the social and environmental conditions of contemporary food production and consumption are expanding the scope of practice and policymaking across the PHN discipline. Malnutrition and poor diets are leading contributors to global morbidity and mortality rates [7]. This has created a double burden of malnutrition, where approximately 1.9 billion people are overweight and obese [87], while more than 805 million are chronically undernourished and 2 billion people suffer from micronutrient deficiencies including iron, iodine, zinc and vitamin A [83,88]. The prevalence of chronic diet-related diseases continues to rise in high-, middle- and low-income countries. In addition, food production impacts on and is impacted by the environment, which in turn effects the amount and types of foods available for consumption [89,90]. The social and geo-spatial patterning of food availability and access, dietary risk exposures and malnutrition have received increasing attention as key contributors to health inequities and the ill-health of vulnerable and marginalised groups [91,92].

Contemporary food systems are increasingly characterised by extensive global supply chains that are removed from local food systems and seasonality through the development of year-round products; high-dependence on chemical-intensive inputs and often bioengineered crops; and the dominant view of agriculture as an ‘input–output process’ that separates the food producer from the consumer [42]. Cannon suggests that modern food production and manufacturing systems have been designed to support the development of nutrition science for the political and economic uses of governments and industry, but have resulted in major food systems and food supply changes [93]. Amplified by competitive pressures from international trade and the consolidation of power and profitability in large corporations within the food producing industry, the intensification of global food production is causing substantial environmental damage and now greatly exceeds natural resource replenishment boundaries [94].

Agricultural activities require more than half of the Earth’s ice-free land and account for around 70 percent of all fresh water extraction [95]. Irrigation practices and the use of chemicals and nitrogenous fertilisers pollute fresh and marine water sources [96]. There has been a rapid decline in arable land due to erosion, salination, acidification, compaction, water-logging and the loss of organic material from over a third of the planet’s fertile soil [97]. Food production practices, such as mono-cropping, also caused extensive biodiversity loss throughout the 20th century [98,99]. The agricultural sector is a significant contributor to climate change, accounting for up to 30 percent of all greenhouse gas (GHG) emissions [100]. Food yields are likely to be affected by climate change as a result of temperature fluctuations and disrupted rainfall patterns, in addition to the damage caused by increased droughts, fires, floods, storms and sea-level rise [13]. The globalization of agriculture has contributed to the tripling of total food production since the 1960s and is expected to double again by 2050 [94]. While increased efficiency has allowed food production to exceed population needs, around one-third of food produced for human consumption is lost or wasted each year [101].

The lack of attention and importance given to the broader impacts of food and nutrition systems has been a constant criticism of the dominant reductionist approach to nutrition science and the productionist paradigm of the food system since the 1930s [102]. Although reductionism has enabled significant advances in nutrition science from a biological perspective, nutrition is well positioned to become a more ‘cross-disciplinary, integrationist life science’ [59], encompassing agricultural, ecological, biological, animal, human, clinical, environmental, political and social sciences in order to meet the PHN challenges of the 21st century [103,104]. The epistemological approach of holism that has been accepted in the development of FBDGs has been extended from a whole-of-organism view of systems, to a much wider consideration of the entire food and nutrition system including both production and consumption. Lang and Heasman similarly describe this approach as the ‘ecologically integrated paradigm’ [102]. These developments are examined below. 

“Nutrition ecology” was developed at the University of Giessen in the mid-1970s by Leitzmann and colleagues [105] and was popularized by Gussow’s *The Feeding Web* published in 1978 [106]. The term ecology was first used by Haeckel in the 1800s and was later interpreted as the study of interrelations, including the exploration of interactions between components of a system and of the components within the wider social and natural environment [107]. Therefore, nutrition ecology simultaneously considers and gives equal importance to health, environment, society, and economy within the food and nutrition system. Nutrition ecology deals with the complexity and multidimensionality of PHN problems by focusing on the problem-oriented integration of knowledge from different disciplines at both a conceptual and methodological level [107]. Hoffman suggests that a distinction should be made between multidisciplinarity, interdisciplinarity and transdisciplinarity in nutrition science and that the discipline should strive for the latter to attain a more holistic paradigm, characterized by the removal of barriers and boundaries between disciplines and institutions in order to achieve true integration [58]. The development of a more ecologically-orientated nutrition coincided with the emergence of sustainability internationally [107,108], recognising that resources should only be used in so far as they can be regenerated [109]. 

Sustainable diets emerged as a means for improving both environmental and human health during the 1980s but were granted little attention as a result of the broader PHN focus on addressing hunger and malnutrition internationally [110]. Also working from the University of Giessen (where von Liebig had originally founded biochemistry, leading to the mechanisation of nutrition science), Koerber and colleagues presented “wholesome nutrition”—a mainly plant-based diet, where minimally processed foods are preferred—as a component of more sustainable nutrition approaches [109]. Gussow and Clancy first proposed a set of dietary guidelines for ES in 1986, highlighting that existing nutrition guidance that supported good health could also support environmental gains [111]. The concept was neglected until the FAO developed a definition that has since gained widespread acceptance and use, acknowledging the interdependencies between human and ecological health, as well as food production and consumption with nutrition guidance. “Sustainable Diets are those diets with low environmental impacts which contribute to food and nutrition security and to healthy life for present and future generations. Sustainable diets are protective and respectful of biodiversity and ecosystems, culturally acceptable, accessible, economically fair and affordable; nutritionally adequate, safe and healthy; while optimizing natural and human resources” ([110], p. 1).

In 2005, Leitzmann and Cannon established the New Nutrition Science Project in direct response to the known limitations of conventional nutrition science. The Giessen Declaration, a major outcome document of the New Nutrition Science Project, established a set of agreed principles, definitions and dimensions for this new paradigm. Under this approach, nutrition science is defined as “the study of food systems, food and drinks and their nutrients and other constituents; and of their interactions within and between all biological, social and environmental systems’ in order to ‘contribute to a world in which present and future generations fulfil their human potential, live in the best of health, and develop, sustain and enjoy an increasingly diverse human, living and physical environment” ([3], p. 786). Recognising these broader dimensions, which reductionist analyses would view as confounding factors, is integral to explaining the diet-health relationship under this paradigm [42]. 

The conceptual development of the ecological paradigm was further expanded through the introduction of a planetary health approach [112], described as “the highest attainable standard of health, wellbeing and equity worldwide through judicious attention to the human systems—political, economic, and social—that shape the future of humanity and the Earth’s natural systems that define the safe environmental limits within which humanity can flourish” ([113], p. 1978). In 2015, UN member states endorsed the Sustainable Development Goals (SDGs), representing a more comprehensive and ambitious development agenda than the predecessor Millennium Development Goals. Underpinning the SDGs is the central concept that sustainable development encompasses the environmental, social and economic needs of present and future generations, as defined at the UN Conference on Environment and Development in Rio during 1992 [114]. The SDGs established a global agenda through 17 goals and 169 targets including the promotion of sustainable food consumption and production to enable the protection and management of natural resources through the development of integrated food and nutrition policies. Nutrition can be linked to almost all of the SDGs and is likely to play a crucial role in the transformation towards more sustainable societies. In particular, Goal 2 seeks to end hunger, achieve food security and improved nutrition and promote sustainable agriculture [6]. This integrated agenda is further acknowledged in the Rome Declaration on Nutrition and its associated Framework for Action agreed to by UN member states at the Second International Conference on Nutrition [115], and within the work programme of the United Nations Decade of Action on Nutrition [116]. These provide important access points for FBDGs that consider ES considerations.

The importance of integrating ES considerations into these and other PHN policy responses is now being recognised [35,110,117]. Of note is the EAT–Lancet Commission on Food, Planet and Health’s landmark 2019 report, highlighting the threat that unsustainable diets and food systems pose for human and planetary health. Importantly, the report defines safe operating boundaries for food production in relation to health and the environment, sets out scientific targets for achieving healthy and sustainable food systems, and defines a universal reference diet that meets nutritional requirements and promotes health within planetary boundaries to minimise damage to Earth’ systems It argues for a “Great Food Transformation” in order to sustainably nourish a projected world population of 10 billion people by 2050 [118]. Achieving this ambition presents a formidable challenge. For example, the seemingly straightforward task of integrating ES considerations into dietary guidelines has been highly contested and politicized, resulting in much variation in the content and purpose of guidelines around the world. Brazil, Canada, Qatar, Sweden and Germany have successfully developed and implemented national dietary guidelines that include or emphasise the central role of sustainable food systems [119,120,121,122]. Clear attempts were made to incorporate ES into the most recent editions of dietary guidelines in Australia and the United States [123,124]. Despite rigorous, evidence-based reviews and consideration by expert committees, both failed to achieve official endorsement as a result of political decisions that indicated a lack of support or the prioritisation of other concerns by the government [35]. In both countries, significant industry interference (e.g., public media campaigns, lobbying) was found to have influenced this outcome [35]. This suggests that although the processes of shifting nutrition guidance towards more systems-based thinking have begun, they are not yet receiving sufficient political support or attention in the face of organized and concentrated opposition [117,125]. 

While there is no clear consensus around the exact nature of dietary guidelines that include ES, some broad principles are acknowledged in current examples. Plant-based diets tend to be more advantageous for health and the environment, especially when compared to the high environmental impact of meat production and its role as a risk factor in many diet-related diseases. Limiting excess energy consumption and discretionary food choices are also recommended in order to avoid both dietary imbalances and the burden they place on the natural resource base, biodiversity and GHG emissions [14]. The Brazilian dietary guidelines also recognise social and economic aspects of sustainability, particularly the way that the expanding consumption of ‘ultra-processed’ foods can displace traditional foods and food cultures [35,119]. Ultimately, none of the original recommendations proposed by Gussow and Clancy have been ‘rendered irrelevant by time’ [126]. However, there is substantial resistance to the integration of ES considerations in FBDGs and other food and nutrition policies [125]. 

It has been the emerging evidence base associated with relationships of foods, food groups and dietary patterns with ES that has been particularly controversial, as debates have encompassed issues about not only the quality of the evidence base but also the purpose of dietary guidelines. While this indicates that the reductionist approach to nutrition science (and the evidence that its methods and techniques support) remains dominant, a key principle for the development of dietary guidelines is the identification of the most critical and relevant public health issues [28]. The nature of public health nutrition problems in the 21st century suggests that traditional, biological understandings of food and health may no longer be sufficient to make change and improve nutritional health outcomes for populations. As McMichael concludes, “the essential challenge for nutrition science is to develop new understanding and strategies to enable a balance between promoting, equitably, the health of humans while sustaining the long-term health of the biosphere” ([94], p. 706). This echoes Kuhn, in that a “paradigm shift” or “scientific revolution” occurs when the underlying assumptions of a discipline are re-examined, and a new paradigm is established.

### 3.5. Synthesis

#### The Nature of Public Health Nutrition Paradigms and Paradigm Shifts

Paradigms may be investigated in terms of their particular properties as well as their functions [40]. As Cannon explains, “evidence that new paradigms are needed will not be found within the normal practice of any discipline. New maps are drawn after new territory is brought to light and old territory is seen in a new light” ([93], p. S481). The findings highlight three established eras of PHN progress—the foundation, nutrient deficiency, and dietary excess and imbalance eras. We have also outlined a fourth, emergent phase—the sustainability era. 

Consistent with previous work, this review found that major historical paradigm shifts in PHN were associated with the identification of vitamins in the first half of the 20th century; recognition of the role of individual nutrients in the development of chronic disease in the second half of the 20th century; and recognition of foods and dietary patterns by the 1990s [25,27,32]. This review extends this work in two important ways—it is the first to consider the effect of the ES challenge on nutrition guidance development and pays particular attention to the ideational and contextual aspects of paradigm shifts by drawing on Kuhn’s theory on the structure of scientific revolutions. Here, the emergence of new public health challenges and the influence of wider contextual forces precipitates a shift in the focus of nutrition science as researchers seek to understand and address the emergent problems. This new knowledge expands existing notions and creates new ways of thinking about nutrition and health, amounting to a paradigm shift consistent with Kuhn’s theory. Hence, the nature and scope of PHN we observe today comprises a series of layered paradigms, of which the most recent is ES. The science underpinning each of the most recent three PHN eras has been communicated through the development of three systems of nutrition guidance—nutrient reference standards, dietary goals and dietary guidelines. 

This paradigmatic approach to understanding PHN has implications for future science, policy and practice. Cannon has previously questioned whether the dominant PHN paradigms have really met their intended purpose of maintaining and improving health, given the mismatch between fundamental concepts of nutrition science and the health and ES outcomes we observe today [93]. New scientific enterprises have recently emerged calling for radical new and integrated ways of approaching the science of food, nutrition and sustainability (e.g., the International Panel of Experts on Sustainable Food Systems (IPES-Food), and the EAT–Lancet Commission on Food, Planet and Health). Others draw to attention to implications of underlying political economies of nutrition, including the power of the food industry in shaping PHN science, policy and practice. Scrinis and Clapp articulate how ‘nutritionism’ (defined as “a reductive focus on the nutrient composition of food”) has enabled the co-option of nutrition science by the food industry ([127,128], p. 41). They argue that reductive nutrition science has enabled ‘functionalization, fortification, and reformulation’ as core features of the commercial marketing of ultra-processed foods, thus promoting dietary patterns inconsistent with public health. Others have drawn attention to the food industry’s long-term funding of nutrition science with major adverse implications for PHN guidance [129,130]. This includes the sugar industry’s role in shifting the focus of PHN science away from sugar and towards cholesterol and fat as dietary causes of coronary heart disease [130]. Important paradigmatic tensions are now also playing out in relation to underlying scientific approaches to food classification and its relationship to nutrition policy and regulation. Specifically, nutrient profiling is currently the dominant approach used to determine the healthiness of foods in policy actions including front-of-pack labelling and health claims, reformulation, food marketing and related regulatory activities [131]. This contrasts with food-based classification schemas that are currently informing nutrition science and guidance, but not policy actions (yet) in some countries. For example, the NOVA classification scheme classifies foods by extent of processing and is now informing dietary guidance in Brazil [132]. 

**Figure 1:**
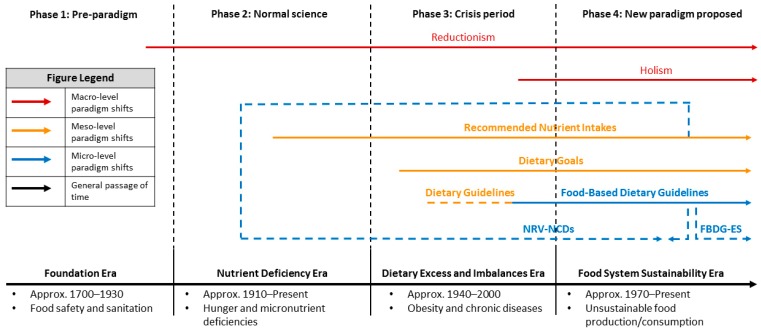
Layering of public health nutrition paradigms. NRV-NCDs = nutrient reference values – noncommunicable diseases; FBDG-ES = food based dietary guidelines – environmental sustainability.

Currently, PHN is undergoing its latest paradigm shift related to increasing awareness of its relationship with ES. In this new phase of PHN, an ecological paradigm has been proposed and has the potential to transform PHN science. However, it is as yet unclear whether this competing paradigm will establish dominance over or continue to evolve alongside more traditional approaches and what effect this will have on the evolution of PHN guidance. Interestingly, we now see a number of smaller shifts occurring within the systems of PHN guidance as a result of diminishing returns from current approaches to address obesity and NCDs and in response to the ES challenge. We therefore propose that the development of PHN is characterized by the successive layering of paradigms and that this may be a result of the interactions between science, social change and policy-making in this discipline, especially in the development of nutrition guidance (See Figure 1). Macro-level paradigms and paradigm shifts are related to the epistemology of nutrition science that underpins the PHN practice and policy-making. Meso-level paradigms reflect the varying emphasis given to nutrients, foods and diets, or the food system as they relate to nutrition-related problems and the potential to solve them through the development of nutrition guidance. Paradigm shifts at this level are represented by the emergence of new systems of nutrition guidance, including nutrient reference values, dietary goals, and dietary guidelines. Micro-level paradigm shifts occur within the various systems of nutrition guidance and relate to the evolution of their intended purpose and scope.

In terms of limitations, this paper does not review the literature around the development of personalised nutrition. Technological advances are creating new possibilities for nutrition research at molecular and genomic levels and may lead to the development of dietary recommendations tailored to an individual’s genetic, metabolic and environmental profile in order to prevent and manage chronic diseases [133]. Several studies of this nature have framed personalised nutrition as the next frontier for nutrition science and guidance [25,27]. We propose that PHN guidance is fundamentally designed for healthy populations. Therefore, personalised nutrition guidance was considered outside of the scope of this review. The literature synthesised by this review also tended to focus on PHN and nutrition guidance in developed countries given that nutrition guidance has advanced more rapidly in these settings, whereas middle- and low-income countries have been acknowledged with respect to the global nature of many of the emerging PHN challenges. Important reviews on the science, policy and politics of nutrition in these contexts exist [134,135], and any future studies on the nature and scope of PHN should integrate this knowledge. Another important omission from this paper is a human rights approach. The human right to adequate food and health (as components of the global human rights system) has provided a foundational normative framework for food security and nutrition improvement in many low- and middle-income country contexts, particularly in Latin America [136]. With some exceptions [137], this approach is under-represented in PHN research, guidance and policy in high-income country contexts, and represents an important consideration for the future of PHN. 

Historical research provides an important tool for advancing our understanding of contemporary PHN challenges. These challenges are particularly complex, multifaceted and value-laden because food and nutrition are central to our physiology, psychology, society and culture. As a result, there are significant implications of PHN practice and policy for economics, politics and power [138]. Our investigation of the nature and dynamics of the PHN science paradigm transitions of the past help to explain some of the recent developments and significant variations in PHN guidance internationally.

In this synthesis, we have traced the development of PHN through four eras of improvement and the relationships between these. There is a significant degree of interactivity between each era because the threat of each prominent PHN challenge—food safety and sanitation, nutrient deficiencies, dietary excess and imbalances, and food system sustainability—can never be completely eradicated. Scientific evidence, as well as learnings from practice and policy-making arising from the new ways of thinking about health and nutrition in an attempt to address increasingly more complex PHN challenges, accumulates over time. While they do not exert the same level of impact as in the era in which they first emerged, these forms of knowledge continue to be important to support the nutrition-related health and wellbeing of populations in the future. These findings are consistent with similar studies on the development of the public health discipline [139,140].

This is mirrored by the successive layering of PHN paradigms and paradigm shifts at three levels—macro, meso, and micro (see Figure 1). PHN paradigm shifts are often characterised by periods of resistance or conflict, usually from segments of the food industry and especially in relation to the purpose and scope of PHN guidance. This is particularly evident when the body of scientific evidence and new ways of thinking are just emerging. It is not until these ideas become more engrained and established that this resistance can be overcome. Callaway’s work suggests that, in this context, initial resistance has historically been “superseded by widespread understanding of the positive outcomes derived from the cooperation of food producers and retailers, public health professionals, federal and state regulatory agencies, and consumers” ([27], p. 2). Kuhn also explains that early denial of a new paradigm is common, but as experiments, instruments and literature increases, more scientists adopt the new paradigm, and a period of normal science returns under its dominance. As such, Kuhn’s notion of paradigm is inherently political, making it appropriate for examining conceptual models of political phenomena [40]. By this logic, it could be hypothesized that the current resistance to integrating ES consideration into FBDGs may ultimately be overcome as the New Nutrition Science paradigm progresses.

Under the dominant epistemological approach of reductionism, current systems of PHN guidance have had limited success in improving nutrition-related health and wellbeing. While it is as yet unclear whether a period of “normal science” will return under the dominance of New Nutrition Science, there is potential for this new way of thinking to bring significant change in and advancement of the PHN field through the development of new scientific tools or methods and more holistic policies. The application of systems-thinking to broader food and nutrition policy planning, development and evaluation has not been addressed fully in this review, due to the focus on systems of PHN guidance. Caraher and Coveney have criticised the tendency of PHN to confine itself mainly to dietary guidelines, rather than engaging with ‘upstream’ policy approaches [86]. Examples of policies that may emerge under this approach include the development of national food plans, which integrate the goals of both food and nutrition policies and therefore consider the food system as a whole [125].

Further analysis of the nature and drivers of paradigm shifts in PHN are limited by the scope of this review. Drawing on Kuhn’s theory of scientific revolutions and paradigm shifts to understand how and why these processes occur, we hypothesise that transitions from one era of PHN nutrition and changes in the scope and content of nutrition guidance over time are triggered by the growing and changing scientific evidence base that supports nutrition science; are politicised by the actions and engagement of policy actors; and are a response to the broader political, social and economic events of the day, leading to a paradigm shift in public health nutrition practice and policy-making. These factors should be considered in future research.

## 4. Conclusions

This review has synthesised the extensive body of literature associated with the historical development of PHN, its scientific underpinnings and the series of paradigm shifts that have contributed to the evolution of PHN guidance over time, culminating in the emergence of a new, transformative paradigm. Major historical paradigm shifts in PHN have been the transition from the foundation era of nutrition to the identification of vitamins in the first half of the 20th century and then the transition to the recognition of the role of foods and dietary patterns in the development of diet-related chronic diseases in the second half of the 20th century. Currently, PHN is undergoing its latest paradigm shift related to increasing awareness of the relationship between PHN and ES and mediated through food systems. Critically, we have identified the dynamics associated with the transition from one era of PHN progress to the next, as largely consistent with Kuhn’s theory on the structure of scientific revolutions. We propose that the development of PHN is characterized by the successive layering of paradigms as a result of the interactions between science, social change and policy-making in this discipline. This especially helps to explain some of the recent developments and significant variation in PHN guidance internationally. This review provides a foundation for conducting further analysis of the nature and drivers of PHN paradigm shifts, as well as potential access points for PHN professionals to advocate for further improvements in this discipline. 

## Figures and Tables

**Table 1:** Overview of Kuhn’s five phases of a paradigm shift.

Phase	Description
Phase 1	The “pre-paradigm” phase occurs only once in the beginning of a scientific discipline and is characterised by incomplete or incompatible theories in the absence of a common body of knowledge to draw on. A dominant paradigm develops as actors begin to form a consensus around conceptual frameworks, terminologies, methodologies and modes of inquiry.
Phase 2	“Normal science” begins under the dominant paradigm and will continue as long as there is consensus within the discipline. Anomalies that are difficult to explain under the current paradigm may occur but are usually resolved. Normal science becomes difficult when anomalies continue to accumulate over time, revealing weaknesses in the paradigm.
Phase 3	If the paradigm cannot continue to explain these anomalies, a crisis period occurs. Crises can often be resolved by normal science but there are other times when the efforts of normal science within a paradigm will fail. The discipline enters the next phase.
Phase 4	A “paradigm shift” or “scientific revolution” occurs when the underlying assumptions of a discipline are re-examined, and a new paradigm is established.
Phase 5	The new paradigm establishes dominance and normal science returns. Problems are identified and solved within the parameters of this new approach. A scientific discipline may undergo this process repeatedly, but paradigm shifts do not occur often or easily.

Footnotes: adapted from Carson et al. [40].

**Table 2:** Summary of the main public health nutrition eras.

Timeframe	Public Health Nutrition Era
Approx. 1700–1930	*Foundation era (Phase 1):* Chemical Revolution provided scientific basis for nutrition science. Industrial Revolution led to spread of communicable diseases/food-borne illness; classic public health interventions and early policies around water, food safety and sanitation emerged.
Approx. 1910–present	*Nutrient Deficiency era (Phase 2):* Discovery of vitamins as nutritional basis of diseases including beriberi, pellagra, scurvy and rickets (now understood to be micronutrient deficiency diseases); identification and synthesis of all major vitamins in first half of the 20th century. Nutrient reference values developed for energy intake and selected nutrients in response to hunger and food shortages during the World Wars and Great Depression. Nutrition science underpinned by reductionist epistemology.
Approx. 1940–present	*Dietary Excess and Imbalances era (Phase 3):* Emergence of chronic diseases as a result of dietary excess and imbalances; cohort studies implicated sugar and fat as risk factors, with fat as the main focus of nutrition guidance. Dietary Goals provided quantitative targets for the consumption of particular nutrients to address micronutrient deficiency and chronic diseases. Subsequent development of dietary guidelines (first generation) with continuing emphasis on individual nutrients. Advances in nutrition research indicated that foods and dietary patterns were more important for chronic diseases, leading to the emergence of food-based dietary guidelines (second generation). Obesity and chronic diseases increasingly recognized as contributing to a worldwide “double burden of malnutrition” (co-existence of under- and over-nutrition). Nutrition science begins to integrate a more holistic epistemology.
Approx. 1970–present	*Food System Sustainability era (Phase 4):* Increasing recognition of environmental challenges in addition to little success in curbing obesity and chronic diseases led to the emergence of a “New Nutrition Science” as an alternative to traditional biomedical approaches to nutrition science and guidance. This was supported by an international sustainable development agenda (Millennium Development Goals and successor Sustainable Development Goals) and the emergence of food-based dietary guidelines that integrate ES considerations in some countries and attempts to do so in others.

**Table 3:** Summary of the systems of nutrition guidance.

System of Nutrition Guidance	Summary
Nutrient Reference Values	Quantitative estimates of human requirements for essential nutrients (expressed in weight/day) that are considered to be adequate for meeting the known nutrient needs of healthy populations [41].
	Developed by expert panels who undertake an evaluation of scientific evidence from human and animal experiments, epidemiological studies and national survey data.
	Used as benchmarks for planning, monitoring and evaluating food and nutrition policies and to assess the adequacy of nutrient intakes for both individuals and groups, identifying those most at risk of micronutrient-related diseases [42].
Dietary Goals	General statement of intent used for long-term planning of national public health nutrition (PHN) policies and programs, indicating a direction and general magnitude of recommended dietary change [30].
	Expressed as average national intakes, dietary goals represent quantified targets for selected macro- and micronutrients to support optimal nutritional health and prevent diet-related chronic diseases. May vary among subpopulations due to varying prevalence of over- and undernutrition [41].
	Development relies on description and quantification of national nutrition systems including: food consumption patterns; selection of target groups and priority objectives; points of intervention within the system; and consideration of potential alternatives [30,42].
Dietary Guidelines	Sets of advisory statements to promote overall nutritional wellbeing and to address all diet-related conditions, considering the customary dietary patterns of healthy populations and indicating what aspects should be modified.
	“Food-based” dietary guidelines refer to the expression of the principles of nutrition education mostly as foods. Where they cannot be expressed entirely as foods, they are written in language that avoids technical nutrition science terms.
	Starting point for devising dietary guidelines is relevance to a public health issue rather than an existing gap between the prevailing nutrient intake and a numerical recommended intake for a nutrient [41].
	Intended to establish an agenda for policies and programs in other sectors including health, agriculture, education, communications, food industry, consumers and research [43].
	Development ideally follows a systematic and transparent process informed by the best available scientific evidence and gives due regard to important contextual factors (social, cultural, economic, agricultural, food supply) while managing conflicts of interest [44]. This approach allows for consensus building, which is essential for successful implementation of the developed guidelines [28].
